# Impaired mitochondrial biogenesis is a common feature to myocardial hypertrophy and end-stage ischemic heart failure

**DOI:** 10.1016/j.carpath.2015.09.009

**Published:** 2016

**Authors:** Annalinda Pisano, Bruna Cerbelli, Elena Perli, Maria Pelullo, Valentina Bargelli, Carmela Preziuso, Massimiliano Mancini, Langping He, Matthew GD Bates, Joaquin R Lucena, Paola Lilla Della Monica, Giuseppe Familiari, Vincenzo Petrozza, Chiara Nediani, Robert W Taylor, Giulia d’Amati, Carla Giordano

**Affiliations:** a Department of Radiological, Oncological and Pathological Sciences, Sapienza University of Rome, Policlinico Umberto I, Viale Regina Elena 324, 00161, Rome, Italy; b Department of Molecular Medicine, Sapienza University of Rome, Policlinico Umberto I, Viale Regina Elena 324, 00161, Rome, Italy; c Department of Biochemical Sciences, University of Florence, Viale Morgagni, 50, 50134, Florence, Italy; d Wellcome Trust Centre for Mitochondrial Research, Institute of Neuroscience, Newcastle University, Newcastle upon Tyne, UK; e Forensic Pathology Service, Institute of Legal Medicine, Seville, Spain; f Department of Cardiac Surgery and Transplantation, San Camillo Hospital, Rome, Italy; g Department of Human Anatomic, Histologic, Forensic and Locomotor Apparatus Sciences, Sapienza University, 00161, Rome, Italy; h Department of Biotechnologies and medical and Surgical Sciences, Sapienza University, 00161, Rome, Italy

**Keywords:** HF, heart failure, LV, left ventricular, OXPHOS, oxidative phosphorylation, mtDNA, mitochondrial DNA, MIC, mitochondrial cardiomyopathies, LCM, laser capture microdissection, COX, cytochrome *c* oxidase, SDH, succinate dehydrogenase, TEM, transmission electron microscopy, *PPARα*, peroxisome proliferator-activated receptor alpha *PPARA*, *PGC-1α*, peroxisome proliferator-activated receptor gamma coactivator 1 alpha, *PPARGC1A*, *NPPA*, natriuretic peptide A, *NRF1*, nuclear respiratory factor 1, *ERRα*, estrogen-related receptor alpha *ESRRA*, *TFAM*, mitochondrial transcription factor A, *POLG*, polymerase (DNA directed) gamma, *HPRT1*, hypoxanthine phosphoribosyltransferase 1, SDS-PAGE, sodiumdodecylsulphate–polyacrylamide gel electrophoresis, PVDF, polyvinylidene fluoride, DNPH, 2,4-dinitrophenylhydrazine, DNP, antidinitrophenyl, CAT, catalase, GPx, glutathione peroxidase, NADPH, nicotinamide adenine dinucleotide phosphate, SOD2, superoxide dismutase 2, MDA, malondialdehyde, Cardiac remodeling, Myocardial hypertrophy, mtDNA depletion, Mitochondrial biogenesis, Oxidative stress, Mitochondrial cardiomyopathy

## Abstract

Mitochondrial (mt) DNA depletion and oxidative mtDNA damage have been implicated in the process of pathological cardiac remodeling. Whether these features are present in the early phase of maladaptive cardiac remodeling, that is, during compensated cardiac hypertrophy, is still unknown.

We compared the morphologic and molecular features of mt biogenesis and markers of oxidative stress in human heart from adult subjects with compensated hypertrophic cardiomyopathy and heart failure. We have shown that mtDNA depletion is a constant feature of both conditions. A quantitative loss of mtDNA content was associated with significant down-regulation of selected modulators of mt biogenesis and decreased expression of proteins involved in mtDNA maintenance. Interestingly, mtDNA depletion characterized also the end-stage phase of cardiomyopathies due to a primary mtDNA defect. Oxidative stress damage was detected only in failing myocardium.

## 1 Introduction

Heart failure (HF) is a complex chronic clinical syndrome and a leading cause of morbidity and mortality in industrialized countries worldwide [1]. Central to the pathogenesis of HF is left ventricular (LV) contractile dysfunction, due either to an ischemic insult (i.e., myocardial infarction), or to nonischemic causes (e.g., hypertension, valvular heart diseases, genetic cardiomyopathies, etc.). These insults induce an inexorable series of maladaptive phenomena that manifest clinically as changes in the size, shape, and function of the heart, collectively referred to as *pathological cardiac remodeling*. The initial event in pathologic remodeling is LV hypertrophy, which eventually evolves to LV dilation and decreased contractility [2].

Clinical and experimental studies have shown that both impaired oxidative phosphorylation (OXPHOS) and increased mitochondria-derived oxidative stress are implicated in the pathophysiology of HF [see for a Review [3], [4]. Thus, the causes of mitochondrial dysfunction in HF are the object of intense investigations, in view of possible therapeutic applications [5]. Previous studies have pointed to altered mitochondrial biogenesis as one of the causal mechanisms of OXPHOS dysfunction in cardiac remodeling. Down-regulation of specific genes (i.e., peroxisome proliferator-activated receptor alpha, *PPARA* aliases *PPARα*; peroxisome proliferator-activated receptor gamma, coactivator 1 alpha, *PPARGC1A* aliases *PGC-1α*) involved in energy metabolism modulation and mitochondrial biogenesis has been shown in several experimental models of HF [6], [7], [8], [9], [10], [11]. However, recent works suggest that the mechanisms at play are more complex in human than in animal models. For example, Karamanlidis et al. showed that mitochondrial dysfunction in the failing human heart is attributable to mitochondrial DNA (mtDNA) depletion rather than decreased transcriptional activity of the mitochondrial genome [12] and pointed to altered mtDNA replication and oxidative-dependent mtDNA damage as possible mechanisms. In addition, an unresolved issue with potential therapeutic implications is whether and to what extent the derangement in mitochondrial biogenesis observed in HF is present in the early phase of cardiac remodeling, that is, during compensated hypertrophy.

In the present work, we aimed to evaluate mitochondrial biogenesis and oxidative damage in compensated myocardial hypertrophy and HF in the adult human heart. To this purpose, we compared ultrastructural, biochemical, and molecular features of myocardial samples from patients with sarcomeric hypertrophic cardiomyopathy (HCM) (as a model of compensated myocardial hypertrophy) and end-stage ischemic cardiomyopathy. For further comparison, we looked for changes in mitochondrial biogenesis and oxidative stress in hypertrophic nonfailing and failing hearts associated with a primary mtDNA defects (all patients with mitochondrial tRNA mutations).

## 2 Materials and methods

### 2.1 Patients

All studies conformed to Sapienza, University of Rome Ethical Committee protocols. Hypertrophic, nonfailing, myocardial samples were obtained from septal myectomy procedures performed on patients with sarcomeric HCM with obstructive physiology (HCM group, *n*= 10). LV myocardial samples from patients with end-stage HF (HF group, *n*= 15) were obtained from transplant procedures. We selected only failing hearts with proven ischemic etiology based both on clinical records and gross morphologic findings. We used as controls [nonfailing heart (NHF) group] LV myocardial samples obtained either from donor hearts, which were unsuitable for transplantation (*n*= 5), or from autopsies of subjects who died for noncardiac causes (i.e., traffic accident), obtained from the Forensic Pathology Service, Institute of Legal Medicine of Seville, Spain (*n*= 5, obtained within 4 h from death).

In addition, we analyzed LV myocardial samples obtained either at autopsy (performed within 4 h from death, *n*= 3) or cardiac transplant (*n*= 3) from patients with genetically proven mitochondrial cardiomyopathies (MICs).

### 2.2 Gross analysis, histology, and enzyme histochemistry

All explanted hearts were weighted and photographed. The epicardial coronary arteries were examined, and the presence and degree of luminal narrowing were evaluated. The myocardium was carefully inspected with short axis sections. Myectomy samples from HCM patients were grossly inspected and measured after surgery.

For routine histological analysis, multiple samples from the left ventricle, ventricular septum (VS), and coronary arteries were embedded in paraffin, and 5-micronmeter-thick sections were stained with hematoxylin–eosin, periodic acid–Schiff and Masson trichrome stain.

Myocardial samples from left ventricle and myectomy were collected, avoiding areas of scarring, and immediately snap-frozen in liquid nitrogen-chilled isopentane, both in cryovials for molecular analyses and in Optimal Cutting Temperature Compound embedding compound for enzyme histochemistry and laser capture microdissection (LCM) experiments.

To identify mitochondrial respiratory chain-deficient cardiomyocytes, we performed sequential cytochrome *c* oxidase (COX) and succinate dehydrogenase (SDH) reactions on frozen sections from the left and right ventricles. Combining the histo-enzymatic mitochondrial reactions for COX (brown) and SDH (blue) normally results in a brown precipitate within the cell in the presence of normal COX activity. As a consequence of mtDNA depletion and/or multiple deletions, mitochondrial protein synthesis is impaired, resulting in a significant reduction in COX activity. In contrast, activity of the entirely nuclear-encoded SDH is retained. Thus, cells with mtDNA defects are highlighted as blue, respiratory-deficient cells following the sequential COX/SDH reaction.

### 2.3 Ultrastructural and morphometric analysis

Mitochondrial ultrastructure was studied in myocardial samples by transmission electron microscopy (TEM). One-cubic millimeter myocardial blocks were dissected from three hearts from each study group and immediately fixed in 2.5% phosphate buffered glutaraldehyde. Tissue dehydration and resin embedding and staining were performed as described [13]. Grids were observed with a Zeiss EM 10 (ZEISS Obercocken, Germany) (TEM), and 15 random fields at 8000 × magnification were acquired and stored as TIFF images (Digital Micrograph 3.4TM, Gatan GMBH, Munchen, Germany). Image analysis was performed using ImageJ 64 1.48a (GNU License, National Institute of Health, Bethesda MD, USA). Mitochondria were counted, the perimeter of each organelle was manually traced, and the mitochondrial area was automatically measured. Both the total and the mean cross-sectional mitochondrial area were then obtained for each acquired field. The sarcomere area was also manually measured for each image, and the ratio between total mitochondrial area and sarcomere area was then derived.

### 2.4 Molecular analyses

Prior to each experiment, sections stained with hematoxylin and eosin and Masson trichrome stain were obtained from each frozen myocardial sample for morphological examination to exclude the presence of extensive fibrosis.

Total DNA from LV myocardial tissue was extracted by Wizard Genomic DNA Purification Kit (Promega, Madison, WI, USA). Total RNA was isolated from LV tissue using the SV total RNA isolation kit (Promega, Madison, WI, USA). RNA amount was measured with NanoDrop ND-1000 spectrophotometer (NanoDrop Technologies, Inc. Wilmington, DE, USA), and total RNA was reverse-transcribed to cDNA using System Capacity cDNA Reverse Transcription Kits (Applied Biosystems, Life technologies Italia, MB, Italy) according to manufactures guidelines.

### 2.5 Quantification of mtDNA and LCM analysis

Total DNA was obtained both from myocardial homogenates and from myocytes microdissected by LCM (Leica LMD 7000, Leica Microsystem, MI, Italy). For the latter, serial 5-μm-thick frozen sections were mounted on a polyethylene foil slide and stained with hematoxylin and eosin. Sections were observed under light microscope with a 40 × objective. Small groups of myocytes (5–10 cells) were microdissected by a ultraviolet laser and collected on an adhesive cap of nanotubes as previously described [14]. Samples were digested with proteinase K (25μg/100μl) over night at 37°C, preamplified by TaqMan® PreAmp Master Mix kit (Life Technologies Italia, MB, Italy) according to manufactures guidelines. Absolute quantification of mtDNA was performed by the standard curve method as previously detailed [14]. The technique involves obtaining the ratio of an unknown variable (number of copies of mtDNA) to a known factor (number of copies of a nuclear DNA gene). With each assay, a standard curve for mtDNA and nDNA was generated using serial known dilutions of a vector in which the regions used as template for the two amplifications were cloned tail to tail to have a ratio of 1:1 of the reference molecules [15]. The absolute mtDNA copy number per cell was obtained by the ratio of mtDNA to nDNA values multiplied by two (as two copies of the nuclear gene are present in a cell). Evaluation of mtDNA content in cardiac homogenate was performed by quantitative-real-time Polymerase Chain Reaction (PCR) as described [15] and expressed as mtDNA/nuclear DNA ratio.

### 2.6 Gene expression analysis by quantitative real-time PCR

The relative expression of the following genes was evaluated by quantitative Real Time PCR using TaqMan probe chemistry by means of inventoried and custom 6-carboxyfluorescein (*FAM*)-labeled TaqMan MGB probe (Life Technologies Italia, MB, Italy) according to the manufacturer's instructions (Supplemental Table 1): natriuretic peptide A (*NPPA*), a molecular marker of cardiac hypertrophy; *PPAR-α*, a critical regulator of cardiac oxidative metabolism; *PGC1-α*; nuclear respiratory factor 1 (*NRF1*); estrogen-related receptor alpha (aliases *ERRα*), important regulators of mitochondrial biogenesis; mitochondrial transcription factor A (*TFAM*) and polymerase (DNA directed) gamma (*POLG*), core components of the mitochondrial transcription; and replication machinery. In all samples, the relative expression of each target gene was evaluated with the comparative threshold cycle (ΔCt) method respect to one control (reference sample) and normalized to the hypoxanthine phosphoribosyltransferase 1 rRNA housekeeping gene.

### 2.7 Western blot analysis

Frozen myocardial tissue was homogenized with the Radio-Immunoprecipitation Assay lysis buffer [50-mM Tris–HCl pH8, 150-mM NaCl, 1% NP-40, 0.5% sodium deoxycholate, 1% sodium dodecyl sulphate (SDS), 1-mM phenylmethylsulfonyl fluoride, 10-mg/ml aprotinin, 10-mg/ml leupeptin, and 10 mg/ml pepstatin] and centrifuged at 14000 *g* for 10 min at 4°C. The protein concentration was measured by protein assay (Bio-rad Laboratories s.r.l, Hercules, CA, USA). Equal amount of protein (40 μg) was separated by 12% sodium dodecyl sulphate–polyacrylamide gel electrophoresis (SDS-PAGE) or precast 4–20% Bolt™ Mini Gels (Invitrogen ™, Life Technologies Italia, MB, Italy) and transferred to a polyvinylidene fluoride (PVDF) membrane. The following primary antibodies were used: antimouse monoclonal PGC-1α (Calbiochem, Millipore, MA, USA); antirabbit monoclonal ERRα (Abcam, Cambridge, UK); antirabbit polyclonal *TFAM* (ATLAS antibodies AB, AlbaNova University Center, Stockholm, Sweden); antimouse β-Actin (− Aldrich, St-Louis, MO, USA). Primary antibodies were visualized using horseradish peroxidase-conjugated secondary antibodies (Jackson ImmunoResearch, Laboratories, Inc., USA). Signals were detected by enhanced chemiluminescence (Promega corporation, Madison, WI, USA).

### 2.8 Oxyblot procedures

Total myocardial protein carbonylation was measured using the OxyBlot Protein Oxidation Detection Kit (Chemicon, Millipore, MA, USA) according to the manufacturer's protocol. Briefly, carbonyl groups were derivatized by reaction with 2,4-dinitrophenylhydrazine for 15 min. Dinitrophenyl (DNP)-derivatized proteins were resolved by 12% SDS-PAGE and transferred to a PVDF membrane. Membranes were incubated 1 h at room temperature with anti-DNP primary antibodies (1:150) and then with goat antirabbit IgG (*horseradish peroxidase* conjugated) antibodies (1:300) for 1 h.

### 2.9 Antioxidant enzyme activities

Antioxidant enzymes activities were evaluated as previously reported [16]. Briefly, for catalase (CAT) activity, 70 μg of tissue homogenates was added to 100-mM phosphate buffer, pH 6.8, containing 10-mM H_2_O_2_. CAT activity was determined following the decrease in absorbance at 240 nm of H_2_O_2_ for 30 s at 25°C [17]. The activity was calculated using an H_2_O_2_ extinction coefficient of 43.6×103 M−1×cm^− 1^and was expressed as μmol/min/mg of protein.

For glutathione peroxidase (GPx) activity, 70 μg of tissue homogenate was added to 100-mM phosphate buffer, pH 7.4, containing 0.5-mM EDTA, 1.0-mM NaN3, 0.25-m MNADPH, 2.25-m MGSH, and 1.0-U/ml glutathione reductase. After addition of 0.24-mM tert-butil hydroperoxide (Sigma-Aldrich, St-Louis, MO, USA), the change in optical density of Nicotinamide adenine dinucleotide phosphate (NADPH) was monitored spectrophotometrically at 340 nm for 2.0 min at 25°C [18]. GPx activity was calculated using a molar extinction coefficient for NADPH of 6.22×103 M−1×cm−1 and expressed as nmol/min/mg protein.

Superoxide dismutase 2 (SOD2) activity was evaluated in 10 μg of tissue homogenate using a Chemical Superoxide Dismutase Assay kit (Cayman Chemical, Ann Arbor, MI, USA) as described [18]. Enzymatic activity was expressed as units/mg of protein.

### 2.10 Lipid peroxidation

Oxidative stress level was evaluated by measuring malondialdehyde (MDA) concentration, as a marker of lipid peroxidation. A Bioxytech LPO-586 (Oxis International Inc. Prodotti Gianni, Milano, Italy) was used.

### 2.11 Statistical analysis

All data are expressed as mean±S.E.M. Data were analyzed by standard analysis of variance procedures followed by multiple pair-wise comparisons adjusted with Bonferroni post-hoc test. Significance was considered at *P*< .05. Numerical estimates were obtained with the Graphpad InStat 3 Version (Graphpad Inc. San Diego, CA, USA).

## 3 Results

### 3.1 Characterization of the study population

Characteristics of the study population are reported in Table 1. Briefly, patients with end-stage heart disease (HF group) were slightly older than patients with HCM and controls (mean age of HF group 57 years, vs. 45 and 43 years of age for the HCM and NHF patients, respectively). All HF patients were either in *New York Heart Association* (NYHA) III or IV while patients with HCM were either in NYHA I or II. The mean ejection fraction (EF) in HF group was lower than 25% while it was within normal limits in HCM and NHF groups, as expected. As molecular markers of myocardial hypertrophy and HF, we evaluated the gene expression level of *NPPA*. As expected, increased levels of *NPPA* were observed in ventricular samples obtained from hypertrophic and failing hearts (Supplemental Fig. 1).

### 3.2 Morphologic features

**Fig. 1:**
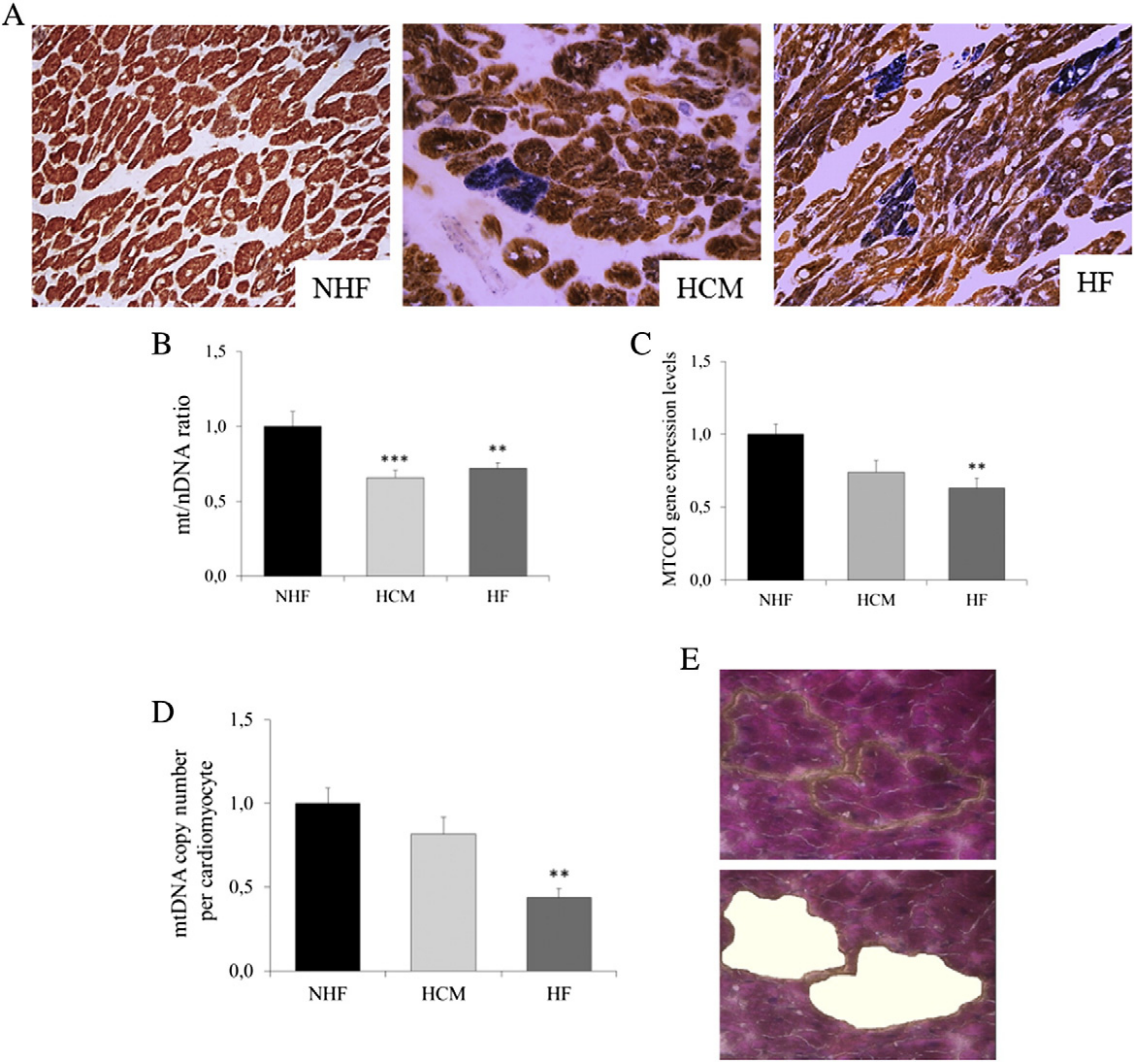
Combined COX-SDH histochemistry and mtDNA amount in myocardium from compensated hypertrophy and end-stage HF. (A) Representative images of combined histochemical reactions for mitochondrial enzymes COX and SDH in NHFs, compensated HCMs, and heart failure (HF). Scattered COX-deficient cardiac myocytes (blue) are observed in both HCM and HF (COX-SDH stain, original magnification × 20). (B) mtDNA amount measured in cardiac homogenate of NHF (*n*= 10), HCM (*n*= 10), and HF (*n*= 15) (expressed as mtDNA/nuclear DNA ratio). Both NHF and HCM show a significant reduction of mtDNA amount as compared to NHF. (C) *MTCO1* messenger RNA relative expression in cardiac homogenate of NHF, HCM, and HF. Both HCM and HF show a reduction in *MTCO1* level as compared to NHF. (D) mtDNA amount in cardiac myocytes microdissected by laser capture from NHF (*n*= 8), HCM (*n*= 6), and HF (*n*= 7) (expressed as mtDNA copy per cardiac myocyte). A statistically significant reduction of mtDNA copies per cardiac myocyte is observed only in HF as compared to NHF. (E) Representative image of myocardium before and after microdissection of single myocytes (hematoxylin and eosin stain, original magnification × 40). Experiments were performed in triplicate. Data are expressed as mean±S.E.M. ^⁎^*P*< .05; ^⁎⁎^*P*< .01; ^⁎⁎⁎^*P*< .001 for HCM and HF versus NHF.

Gross and histologic evaluation confirmed the clinical diagnosis in all explanted hearts from patients with ischemic cardiomyopathy: in 10 cases (66%), there was severe atherosclerosis of the epicardial coronary arteries, complicated by transmural myocardial infarction. In five hearts (33%), there was diffuse intramural coronary artery disease, with multiple small myocardial scars in the absence of significant stenosis of the major epicardial vessels. Surgical samples from myectomy procedures in patients with HCM showed marked myocytes hypertrophy and disarray, along with a mild increase in interstitial collagen and focal microvascular disease, consistent with the diagnosis of sarcomeric HCM. Control hearts were unremarkable, both on gross and pathologic examination. Sequential COX/SDH reactions, performed to identify respiratory chain-deficient cardiac myocytes in the three groups, showed rare, scattered (< 1 per high-power field) COX-deficient cells in the left ventricle of all HF samples and in 8/10 HCM samples. COX-deficient cells were never observed in controls (Fig. 1A).

### 3.3 Hypertrophic and failing heart shows partial mtDNA depletion

As a marker of mitochondrial biogenesis we first analyzed the mtDNA amount of whole heart homogenates in the three groups. We showed a ~ 30% decrease of mtDNA content (expressed as mtDNA/nuclear ratio) both in HCM (*n*= 10) and HF (*n*= 15) as compared with NHF (*n*= 10). This was paralleled by a decreased expression of the mitochondrial gene *MTCOI*, coding for a subunit of complex IV of the respiratory chain (Fig. 1B and C). Since cardiac homogenate is not only composed of cardiomyocytes but also of other tissue components (i.e., fibrosis, vessels, etc.) that may be differently represented in the three study groups, we performed LCM on frozen cardiac sections to selectively measure the mtDNA amount in cardiomyocytes. By this technique, we confirmed the significant mtDNA reduction in HF (57% decrease as compared with controls) while there was a less-pronounced reduction (19%) in HCM (Fig. 1D and E).

### 3.4 Partial mtDNA depletion is associated with reduced expression of proteins involved in mtDNA replication

**Fig. 2:**
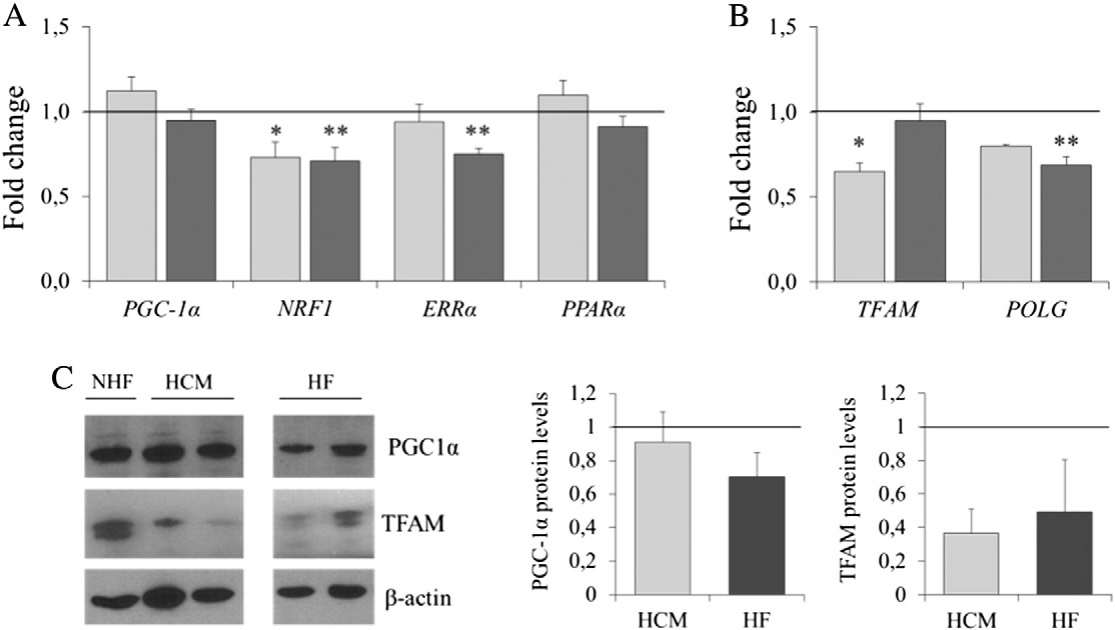
Expression levels of genes and proteins involved in mitochondrial biogenesis. (A) Messenger RNA relative expression levels of *PGC-1α, NRF1*, *EERα*, and *PPARα*. A significant decrease of *NRF1* and *EERα* was found in HF (dark gray) as compared to NHF. *NRF1* levels were significantly decreased also in HCM (light gray) as compared to NHF. Experiments were performed in triplicate. (B) Messenger RNA relative expression levels of *TFAM* and *POLG*. Both genes showed a general reduction, both in HF and HCM as compared to NHF. However, the reduction was statistically significant for *POLG* in HF (dark gray) and *TFAM* in HCM (light gray). Experiments were performed in triplicate. (C) Expression of selected proteins involved in mitochondrial biogenesis and mtDNA maintenance: representative western blot and densitometry are shown. Normalization relative to β-actin (mean of three experiments) showed a generalized reduction of *TFAM* both in HCM and HF as compared to NHF. PGC-1α levels were similar in the three study groups. Data are expressed as mean±S.E.M. and are normalized to the level of NHF (indicated by the line). ^⁎^*P*< .05; ^⁎⁎^*P*< .01; for HCM and HF versus NHF.

We then evaluated that the hypothesis that reduced mtDNA content in hypertrophic and failing hearts could be related to reduced mitochondrial biogenesis. Accordingly, we measured the relative expression levels of genes involved in mitochondrial biogenesis. As shown in Fig. 2A, *PGC-1α*, the master regulator of mitochondrial biogenesis, was unchanged both in HCM and HF, while selected transcriptional regulators that PGC-1α co-activates behaved differently. In fact, both *NRF1* and *ERRα* appeared reduced in both conditions (up to 25% decrease), while *PPARα* levels were unchanged. Finally, reduction of both *TFAM*, the key regulator of mtDNA transcription and mitochondrial *POLG*, was observed both in HCM and HF (Fig. 2B).

Western blot analysis confirmed a clear reduction of *TFAM* protein in both HCM and HF groups while PGC-1α was unchanged. However, possibly because of the small data set and the intrinsic interindividual variability, differences in protein levels between groups did not reach statistic significance (Fig. 2C).

### 3.5 Cardiomyocytes from hypertrophic and failing heart show increased number and reduced size of mitochondria

**Fig. 3:**
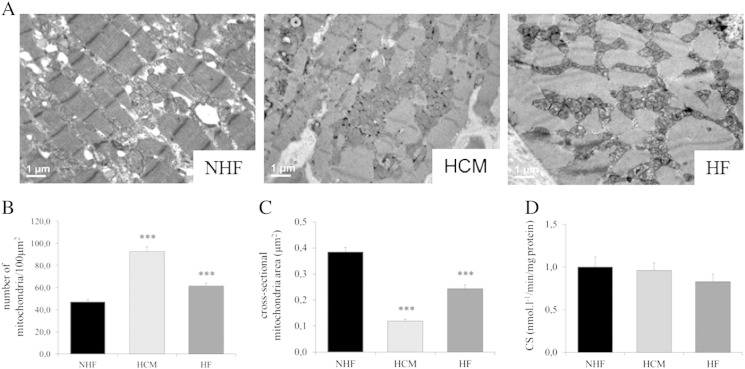
Mitochondrial density and morphology and CS activity. (A) Representative ultrastructural images of NHF, HCM, and HF samples. (B) Graph summarizing the number of mitochondria per area (100 μm^2^) in myocardium of NHF (*n*= 3), HCM (*n*= 3), and HF (*n*= 3). Number of mitochondria is significantly increased both in HCM and HF as compared to NHF. (C) Graph summarizing the average mitochondrial size in myocardium of NHF, HCM, and HF. The mean cross sectional area of mitochondria is significantly decreased both in HCM and HF as compared to NHF. (D) CS activity in cardiac homogenate from NHF, HCM, and HF. Cytrate synthase activity was comparable in the three groups. Data are expressed as mean±S.E.M. ^⁎⁎⁎^*P*< .001 for HCM and HF versus NHF.

We performed ultrastructural morphometric analysis on a subset of myocardial samples (NHF=3; HCM=3; HF=3) to evaluate both density and morphology of mitochondria. A mean of 1251 isolated mitochondria (defined as organelles enclosed by a double contoured membrane) were analyzed from each sample (range, 940–1690; standard deviation±326.16). As shown in Fig. 3A, mitochondria in HCM and HF were increased in number and appeared smaller as compared with controls. These features were more marked in HCM. In fact, the absolute number of mitochondria per area was increased by 96% in HCM and 35% in HF (Fig. 3B) while the mean cross-sectional area of individual mitochondria was reduced by 70% in HCM and 39% in HF, as compared with controls (Fig. 3C). To assess if the observed mitochondrial remodeling in cardiac hypertrophy and failure reflected changes in the total mitochondrial volume mass, we evaluated the activity of citrate synthase (CS), a key enzyme in the Krebs cycle. As shown in Fig. 3D, the activity of CS was unchanged both in HCM and HF.

### 3.6 Markers of oxidative stress and antioxidant enzyme activities are increased only in end-stage failing hearts

**Fig. 4:**
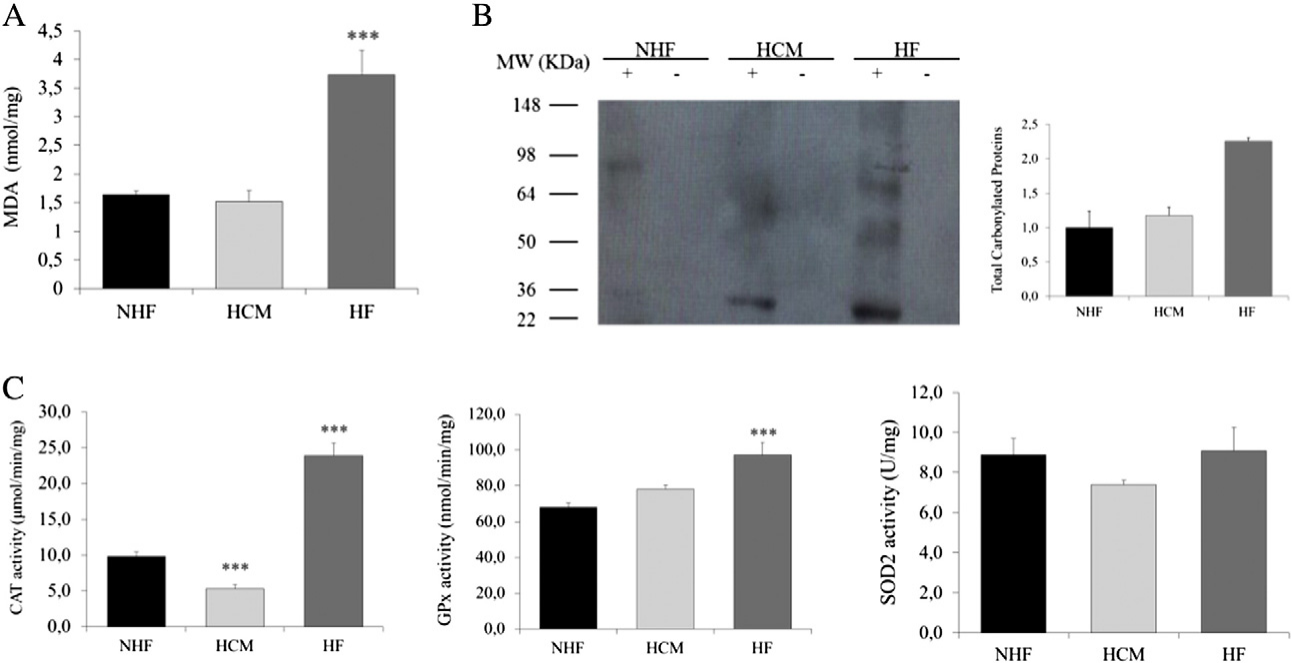
Markers of oxidative stress and antioxidant enzymes activity. (A) MDA levels evaluated on whole homogenate of NHF, HCM, and HF. MDA levels were significantly increased only in HF. (B) Representative western blot analysis and quantification by densitometry of total carbonylated protein from NHF, HCM, and HF. The Ponceau S stained membrane is shown in supplemental Fig. 2A. Carbonylated proteins are increased in HF as compared to NHF. (C) Activities of the antioxidant enzymes CAT (expressed as μmol/min/mg of protein), GPx (expressed as nmol/min/mg of protein), and SOD2 (expressed as U/mg of protein). Only failing hearts showed a significant increase of CAT and GPx activity as compared to NHF. SOD2 activity was comparable in the three groups. Data are expressed as mean±S.E.M. ^⁎⁎⁎^*P*< .001 for HF versus NHF.

Since myocardial oxidative damage has been implicated both in mtDNA damage and in the progression to HF, we looked for markers of oxidative stress in HCM and HF. Both MDA, a terminal product of lipid peroxidation, and protein carbonylation, assessed by oxyblot, were significantly increased only in HF (Fig. 4A and B). We then evaluated the activities of the antioxidant enzymes CAT, GPx, and SOD2. Only failing hearts showed a significant increase of antioxidant enzymes, consistent with increased oxidative stress in this condition (Fig. 4C).

### 3.7 MICs show mtDNA depletion in the end-stage phase.

Genetic and biochemical data of patients with MIC are reported in Table 2. Briefly, Patients 1–4 belong to unrelated families and harbored heteroplasmic levels of the pathogenic m.3243A>G mutation in the mitochondrial gene MTTL1 coding for tRNALeu. The clinical phenotype ranged from encephalomyopathy with lactic acidosis (MELAS = *Mitochondrial encephalomyopathy, lactic acidosis, and stroke-like episodes*) to myopathy and sensorineural deafness. HCM was diagnosed in the course of cardiologic screening. Patient 5 belong to a large family with isolated MIC caused by the homoplasmic m.4300A>G mutation in the mitochondrial gene MTTI coding for tRNAIle (see 21 for a detailed clinical history). Patient 6 [19] showed HCM and progressive hearing impairment. He harbored homoplasmic levels of the pathogenic m.4277C>T mutation in the mitochondrial MTTI gene. Based on electrocardiographic and echocardiographic features (Table 3), patients were divided into two groups, MIC with compensated cardiac hypertrophy (hypertrophic [H] MIC, *n*= 3) and end-stage MIC (HF MIC, *n*= 3).

**Fig. 5:**
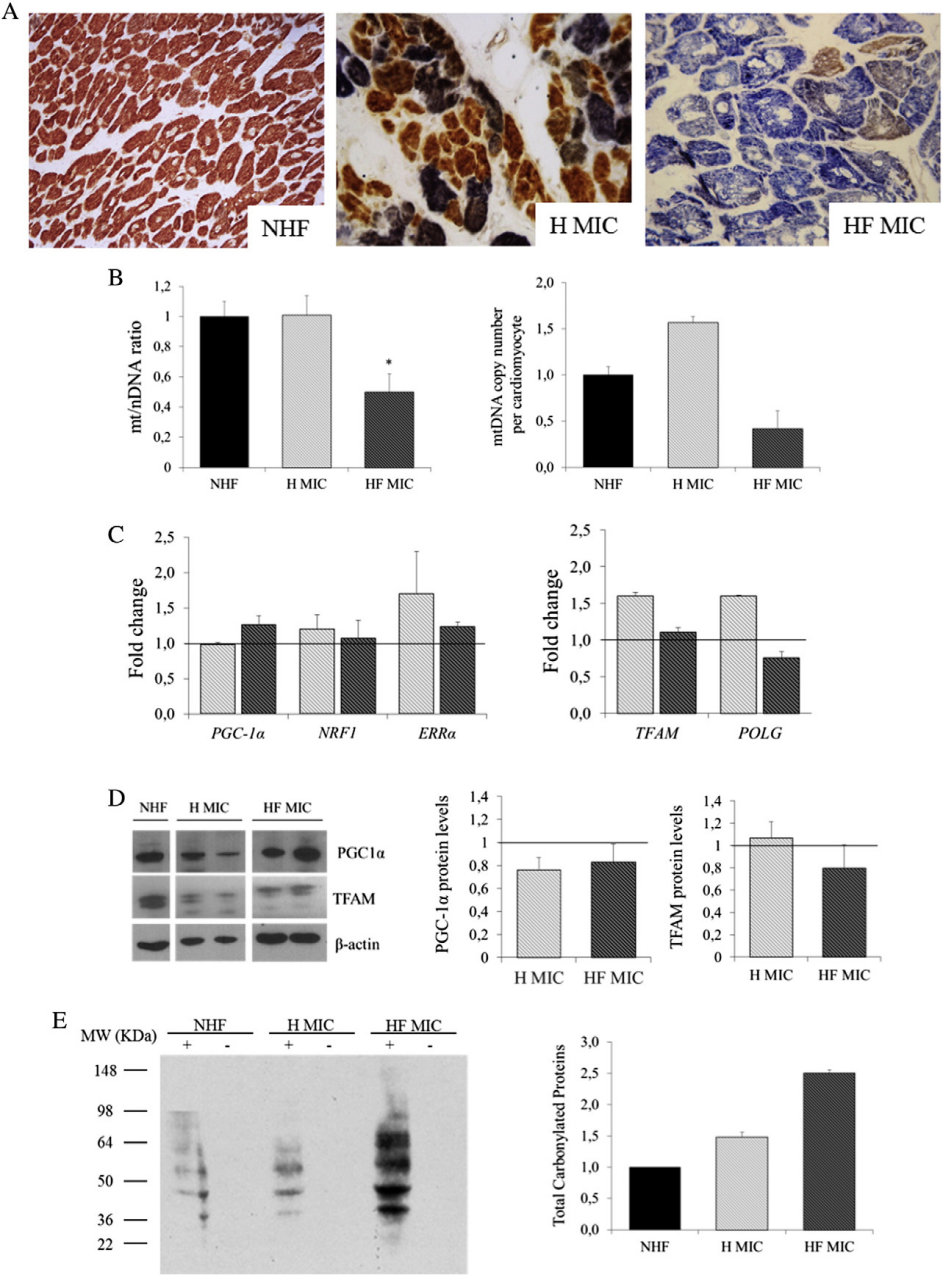
Markers of mitochondrial function and biogenesis and levels of carbonylated proteins in MIC. (A) Representative images of combined histochemical reactions for mitochondrial enzymes COX and SDH in NHFs, MIC with compensated cardiac hypertrophy (H MIC), and end-stage MIC (HF MIC). A large amount of COX-deficient cardiac myocytes is observed both in H MIC and HF MIC. (B) mtDNA amount (mean±S.E.M.) in NHF (*n*= 8), H MIC (*n*= 3), and HF MIC (*n*= 3) evaluated on whole cardiac homogenate (left) and microdissected cardiac myocytes (right). mtDNA depletion was observed in end-stage MIC, while a slight nonsignificant increase mtDNA characterized microdissected cardiac myocytes from H MIC. (C) Messenger RNA relative expression levels of *PGC-1α*, *NRF1*, *ERRα*, *TFAM*, and *POLG* in NHF (*n*= 10), H MIC (*n*= 2), and HF MIC (*n*= 2). Level of *ERRα*, *TFAM*, and *POLG* were slightly increased in H MIC (light gray) as compared to NHF and HF MIC (dark gray). ±S.E.M. and are normalized to the level of NFH (indicated by the line). (D) Expression of selected proteins involved in mitochondrial biogenesis and mtDNA maintenance: representative western blot and densitometry are shown. Densitometry data are expressed as mean±S.E.M. and are normalized to the level of NFH (indicated by the line). (E) Representative western blot analysis and densitometry (mean±S.E.M.) of total carbonylated protein from NHF, H MIC, and HF MIC. The Ponceau S stained membrane is shown in Supplemental Fig. 2B. Carbonylated proteins are markedly increased in HF MIC as compared to NHF.

Consistent with the mitochondrial etiology, combined COX/SDH reactions on frozen heart sections showed a mosaic pattern of COX-positive and COX-deficient cells, with a prevalence of the latter in the presence of mutation levels above 90% (Fig. 5A). The more interesting result, at molecular analysis, was the significant mtDNA reduction in HF MIC as compared with both H MIC and controls. This feature was paralleled by a relative down-regulation of *TFAM* and *POLG* genes as compared to H MIC. Protein levels were unchanged (Fig. 5B–D).

A marked increase in oxidative stress was observed only in end-stage MIC (Fig. 5E).

## 4 Discussion

Despite significant progress in cardiovascular medicine, HF remains a leading cause of morbidity and mortality in industrialized countries. Failing hearts invariably show abnormal energy metabolism, increased reactive oxygen species (ROS) production and mitochondrial respiratory chain dysfunction [20], [21]. These are associated with impaired mitochondrial biogenesis, suggesting that mitochondria-targeted therapies may be effective in HF [22]. Important and unresolved issues are whether impaired mitochondrial function and biogenesis are present already in the early stage of myocardial remodeling, that is, during compensated hypertrophy, and which is the underlying pathogenic mechanism.

Studies on animal models show that deregulation of mitochondrial biogenesis is an early event in HF, and appears to be triggered by down-regulation of the PGC-1α pathway [6], [7], [9], [10]. However, results obtained in animal models have not been verified in human, due to the difficulties in obtaining surgical cardiac tissue samples from patients with compensated hypertrophy. In a previous study, Karamanlidis et al. [23] analyzed mitochondrial biogenesis in myocardial samples from hypertrophic and failing right ventricles of pediatric patients undergoing surgery for congenital heart disease. According to their results, both the hypertrophic and failing ventricles showed a lower mtDNA content as compared to normal hearts, suggesting that mitochondrial dysfunction is involved in both processes of compensated hypertrophy and overt HF. However, the age difference between subjects with HF (median age: 0.8 months), compensated hypertrophy (median age: 6.3 years), and controls (median age: 12 years) could by itself account for some of the observed changes and made results difficult to interpret [24]. The reported higher amount of mtDNA in control subjects, as compared with patients with compensated hypertrophy and HF, could reasonably be due, at least in part, to the induction of mitochondrial biogenesis during heart growth [25], [26].

In the present study, we focused our analysis on adult subjects. We compared mitochondrial biogenesis and markers of oxidative stress in myocardial samples from patients with either sarcomeric HCM (as a model of compensated myocardial hypertrophy) or end-stage ischemic cardiomyopathy, with age-matched controls. We showed that mtDNA depletion and decreased levels of proteins involved in mtDNA maintenance are common features to both conditions, suggesting that impaired mitochondrial biogenesis is an early event in cardiac hypertrophic remodeling. mtDNA depletion in HCM was more evident in the total heart homogenate as compared with dissected cardiac myocytes. A likely explanation for this observation is that the mtDNA content in total heart homogenate derives both from cardiac myocytes and interstitial cells (i.e., fibroblast, endothelial, and smooth muscle cells). In fact, interstitial tissue is invariably increased in HCM [27], [28]. According to their low energy requirement, interstitial cells are expected to have lower mtDNA amount as compared to cardiac myocytes [29], [30]. Thus, the total mtDNA amount in HCM homogenates may be “diluted” by the interstitial cells.

Changes in mtDNA levels were paralleled by down-regulation of the key modulators of mitochondrial biogenesis *NRF1*, ERRα, *TFAM*, and POLG1 but not of PGC-1α, as previously reported in HF [12], [31].

Both in compensated hypertrophy and in HF, the decrease in mtDNA content is paralleled by an increase in the number of intermyofibrillar mitochondria that appear smaller than controls, while the total mitochondrial mass is unchanged. The significance of these features is unclear and should be confirmed in a higher number of samples. Chen et al. [32] recently showed that the increased number of small mitochondria in heart disease is caused by an altered mitochondrial fusion/fission balance and speculated that this may be related to increased myocyte apoptosis, contributing to HF.

Since reactive oxygen species have been implicated as potential modulators of mitochondrial biogenesis [12], we then looked for markers of oxidative damage in myocardial samples from compensated hypertrophy and HF. Lipid peroxidation and protein carbonylation were raised exclusively in the failing myocardium and were associated with activation of the antioxidant enzymes CAT and GPx, while the activity of the mitochondrial antioxidant enzyme SOD2 was unchanged, according to previous observations [33], [18].

All together our results show that impaired mitochondrial biogenesis is a constant feature of cardiac maladaptive remodeling being present both in compensated HCM and in overt cardiac failure. Conversely, oxidative stress damage is detected only in failing myocardium. Thus, oxidative stress damage seems not to be a causative factor of impaired mitochondrial biogenesis and mtDNA depletion in cardiac myocytes, at least in this setting.

We then analyzed mitochondrial biogenesis and oxidative stress in cardiomyopathies caused by mutations in genes coding for mt-tRNAs. These mutations cause structural alteration and reduced stability of mt-tRNAs that affect whole mitochondrial protein synthesis, leading to a respiratory chain dysfunction. The net effect is the reduction in cellular energetic proficiency and the increase in mitochondrial-derived ROS production. A common feature of mitochondrial disease caused by mtDNA mutations is the up-regulation of mitochondrial biogenesis, to overcome the decrease in ATP levels due to respiratory chain dysfunction [34], [35]. Accordingly, we observed induction of mitochondrial biogenesis in the hypertrophic phase of MIC. However, failing MIC showed mtDNA depletion and decrease of genes involved in mtDNA maintenance. Again, marked oxidative stress damage was evident only in the end-stage hearts.

## 5 Conclusion

In conclusion, altered mitochondrial biogenesis, mtDNA depletion, and oxidative stress damage are constant features of HF irrespective of the underlying etiology. The mechanisms involved in deranged mitochondrial biogenesis and mtDNA depletion and the impact of this derangement on the progression of pathological cardiac remodeling still needs to be elucidated. Our results suggest that oxidative stress is not implicated in mediating mtDNA depletion, at least in the early phase of cardiac remodeling.

## Figures and Tables

**Table 1:** Characteristics of patients with compensated HCM and end-stage ischemic heart disease

	NHF (*n*= 10)	HCM (*n*= 10)	HF (*n*= 15)
Age in years, median (range)	43 (17–72)	45 (36–59)	57 (45–67)
Gender, male	7	7	14
EF (%)	58±7#	63±4⁎	24.6±2⁎
Cardiac Index (L/min/m^2^)	na	2.87±0.43	2.28±0.04⁎⁎
PWP, mm Hg	na	13.5±3.34	22.2±2.7⁎⁎⁎
PAP, mm Hg	na	24.8±3.85	33.4±3.3⁎⁎⁎

Treatments			
ACEis	na	0	15
β-blockers	na	3	13
Statins	na	0	3

Associated disease			
Diabetes	na	0	5
Dyslipidaemia	na	0	5
Hypertension	na	3	8

NHF= non failing heart; HCM= hypertrophic cardiomyopathy; HF= heart failure.

na: not available.

# : EF refers only to the five heart donors. EF values were not available for the autoptic cases.

⁎ *P*< .001 vs. NFH.

⁎⁎ *P*< .01 vs. HCM.

⁎⁎⁎ *P*< .001 vs. HCM.

**Table 2:** Genetic and biochemical features of MICs

Patient	Phenotype	mtDNA mutation	% of mutation on heart homogenate	Combined COX/SDH	*NPPA* (mean±S.E.M.)
1	MELAS, diabetes	m.3243 A>G *MTTL1*	77%	Scattered COX-negative fibers	4.96±1.18
2	MELAS	m.3243 A>G *MTTL1*	92%	Prevalence of COX-negative fibers	5.6±0.22
3	MELAS	m.3243 A>G *MTTL1*	76%	na	na
4	Cardiomyopathy, myopathy, CPEO, deafness	m.3243 A>G *MTTL1*	89%	Prevalence of COX-negative fibers	na
5 (Patient IV-09 in Taylor et al. 2003)	Isolated MIC	m. 4300 A>G *MTTI*	Homoplasmic	Prevalence of COX-negative fibers	62.18±0.54
6 (Perli et al. 2012)	Isolated MIC	m.4277 C>T *MTTI*	Homoplasmic	Prevalence of COX-negative fibers	28.12±0.8

MELA: Smitochondrial encephalomyopathy, lactic acidosis, stroke-like episode.

na: not available.

CPEO: chronic progressive external opthalmoplegia.

**Table 3:** Clinical features of MIC patients

	Gender	AD	Cause of death	Electrocardiogram				Echocardiogram					Medication
				Rhythm	LVH	Strain	Other	EF%	LVEDD	LVESD	LVWT VS	LVWT PW	
H MIC													
1	M	35	SD	Sinus	Yes	Yes	RVH	N	N	N	16	15	BB, ACEi
2	M	45	SD	Sinus	Yes	Yes	No	45	N	N	N^1^	N^1^	ACEi
3	M	36	SD	Sinus LBBB	Yes	No	Short PR	55	44.5	34.5	12.5	9.5	BB, ACEi

FH MIC													
4	M	30	CHF^2^	Sinus, biventricular PM	No	No	No	15	62	59	N^1^	N^1^	Dobutamine
5	M	23^3^	CHF	Sinus	Yes	Yes	TWI	17	82	72	25	16	BB, ACEi
6	M	20^3^	CHF	Sinus	Yes	Yes	TWI	25	69	63	21	18	BB, ACEi

AD: age at death.

LVH: left ventricle hypertrophy.

LVEDD: left ventricular end-diastolic diameter.

LVESD: left ventricular end-systolic diameter.

LVWT: left ventricular wall thickness.

PW: posterior wall.

SD: sudden death.

RVH: right ventricle hypertrophy.

BB: beta blockers.

ACEis: ACE inhibitors.

LBBB: left bundle brank block.

PR: PR interval.

CHF: congestive heart failure.

PM: pace maker.

TWI: T-wave inversion.

N: normal.

1 Upper limit of normal.

2 Sudden death while waiting for cardiac transplant.

3 Cardiac transplant.
