# Supplementary material for: Impaired mitochondrial biogenesis is a common feature to myocardial hypertrophy and end-stage ischemic heart failure
Source: Cardiovasc Pathol. 2016 Mar-Apr;25(2):103–12. doi: 10.1016/j.carpath.2015.09.009 (PMC4758811; doi:10.1016/j.carpath.2015.09.009)

**Supplemental table 1**. Real-time PCR assays used to assess gene expression levels.

| **GENE** | **Assay ID or primers and probe sequences** |
| --- | --- |
| *HPRT1* | Hs 99999909_m1 |
| *NPPA* | Hs 00383231_m1 |
| *PPARα* | Hs 00947537_m1 |
| *PGC-1α* | Hs 01016721_m1 |
| *NRF1* | Hs 00192316_m1 |
| *ERRα* | Forward: 5’- CCTAGAGATTGTGGTCACCAT-3’  Reverse : 5’- ACACGCTCTGCAGTACTGACATC-3’  Probe: VIC- CATCCCAGGCTTCT-MGB |
| *TFAM* | Hs 01082775_m1 |
| *POLG* | Hs 00160298_m1 |
| *MTCOI* | Hs 02596864_g1 |

**Supplemantal Figure 1.** **NPPA gene expression levels in myocardial hypertrophy and failure.**

NPPA gene expression levels in non failing hearts (NHF), hypertrophic cardiomyopathy (HCM) and failing hearts (HF). Both HCM and FH show increased expression of NPPA as compared to NHF. (*p<0,05,***p<0,001 for HCM and HF *versus* NF).


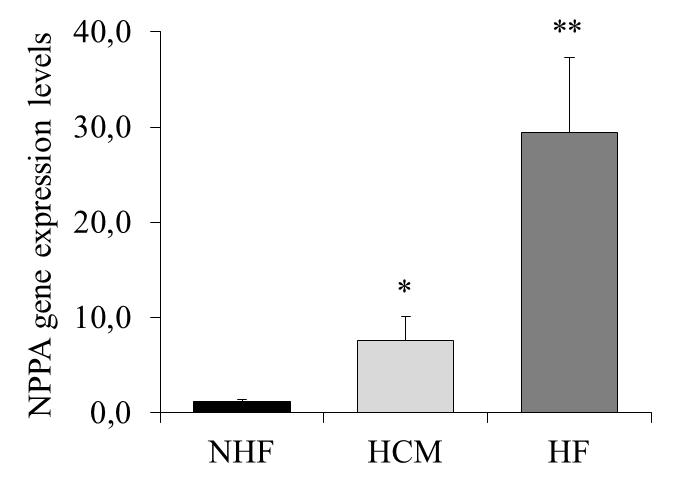


**Supplemental Figure 2.** Ponceau S stained membranes relative to the oxyblots showed in Figure 4B and 5E


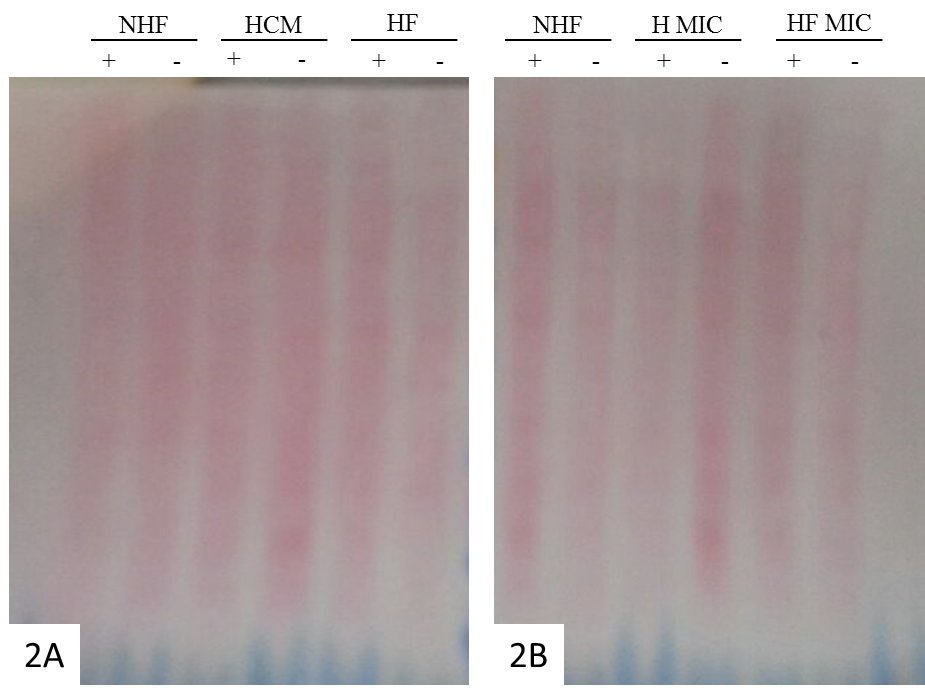

Supplement: Supplementary file 1 — Supplementary material. [file mmc1.docx]
